# Strong signature of electron-vibration coupling in molecules on Ag(111) triggered by tip-gated discharging

**DOI:** 10.1038/s41467-023-41601-2

**Published:** 2023-09-25

**Authors:** Chao Li, Christoph Kaspar, Ping Zhou, Jung-Ching Liu, Outhmane Chahib, Thilo Glatzel, Robert Häner, Ulrich Aschauer, Silvio Decurtins, Shi-Xia Liu, Michael Thoss, Ernst Meyer, Rémy Pawlak

**Affiliations:** 1 Department of Physics, University of Basel, Klingelbergstrasse 82, 4056 Basel, Switzerland; 2 Institute of Physics, University of Freiburg, Hermann-Herder-Strasse 3, 79104 Freiburg, Germany; 3 Department of Chemistry, Biochemistry and Pharmaceutical Sciences, University of Bern, Freiestrasse 3, 3012 Bern, Switzerland; 4 Department of Chemistry and Physics of Materials, University of Salzburg, Jakob-Haringer-Strasse 2A, 5020 Salzburg, Austria; 5 EUCOR Centre for Quantum Science and Quantum Computing, University of Freiburg, Hermann-Herder-Str. 3, 79104 Freiburg, Germany

**Keywords:** Scanning probe microscopy, Molecular electronics

## Abstract

Electron-vibration coupling is of critical importance for the development of molecular electronics, spintronics, and quantum technologies, as it affects transport properties and spin dynamics. The control over charge-state transitions and subsequent molecular vibrations using scanning tunneling microscopy typically requires the use of a decoupling layer. Here we show the vibronic excitations of tetrabromotetraazapyrene (TBTAP) molecules directly adsorbed on Ag(111) into an orientational glassy phase. The electron-deficient TBTAP is singly-occupied by an electron donated from the substrate, resulting in a spin 1/2 state, which is confirmed by a Kondo resonance. The TBTAP^•−^ discharge is controlled by tip-gating and leads to a series of peaks in scanning tunneling spectroscopy. These occurrences are explained by combining a double-barrier tunneling junction with a Franck-Condon model including molecular vibrational modes. This work demonstrates that suitable precursor design enables gate-dependent vibrational excitations of molecules on a metal, thereby providing a method to investigate electron-vibration coupling in molecular assemblies without a decoupling layer.

## Abstract (web summary)

Electron-vibration coupling is driving advances in molecular electronics, spintronics, and quantum technology. Here, the authors succeeded in directly controlling vibronic excitations in tetrabromotetraazapyrene (TBTAP) molecules on the surface of Ag(111).

## Introduction

Electron–vibration (*e*–*ν*) coupling is a central topic in a wide range of molecular electronics, spintronics and quantum computing. The *e*–*ν* coupling in a single-molecule junction strongly influences charge transport through the molecule^1–10^. When an electron tunnels from the electrode onto a molecule, the molecule enters a charge state that can force the relaxation of the nuclear coordinates. This process, which gives rise to vibrational excitations of a molecule during an electronic transition, is commonly treated within the Franck–Condon (FC) picture^11,12^. In this model, an instantaneous electronic transition is assumed, in contrast to the slow motion of nuclei that is directly visible in the low-temperature transport spectroscopy of single molecules^1,13–16^. However, detecting the *e*–*ν* coupling is a difficult task because it requires to control down to the nanometer scale, where such electronic transitions only have femtosecond lifetimes^17–20^.

Scanning tunneling microscopy (STM) and spectroscopy (STS) are useful tools for this purpose, as they can visualize the topography of molecules on surfaces at the atomic scale and probe their electronic states. The strong *e*–*ν* coupling, whose fingerprint is a sequential electron transport during charge or discharge processes in single-molecules, has been measured using STS on various substrates^4,5,21,22^, but also in transport devices^23–26^. While the study of the charge-state control and vibrational excitations of molecules on decoupling layers was widely reported^27–34^, observing *e*–*ν* coupling in STS measurements is more challenging when molecules are directly adsorbed on metal surfaces^35^.

Despite the high lateral resolution of a STM, the short lifetime of charge states in molecular species in direct contact with a metal substrate typically results in a broadened peak in the differential conductance, making the detailed observation of vibrational excitations challenging^4,35^. This problem can be circumvented by decoupling molecules from the metal using an insulating layer to better resolve them with high intensity and resolution^22,27,36–38^. However, exploring vibrational excitations in molecular assemblies on decoupling layers is rather scarce in the literature since assembly protocols are less established than those on metals.

As a consequence, a facile protocol has been developed through the judicious design and chemical modification of a pyrene core. The insertion of four nitrogen atoms at 1-,3-,6- and 8-positions of the aromatic ring imparts a strong electron-deficiency to tetraazapyrene (TAP)^39,40^. The subsequent introduction of four bromine atoms at 4-,5-,9- and 10-positions of the TAP core is beneficial to multiple types of non-covalent intermolecular interactions, leading to the formation of N ⋯  Br and Br ⋯  Br halogen bonds and N ⋯  H and Br ⋯  H hydrogen bonds^41–43^. On the other hand, the bulky bromine atoms can efficiently decouple the TAP core from a metal surface.

**Fig. 1:**
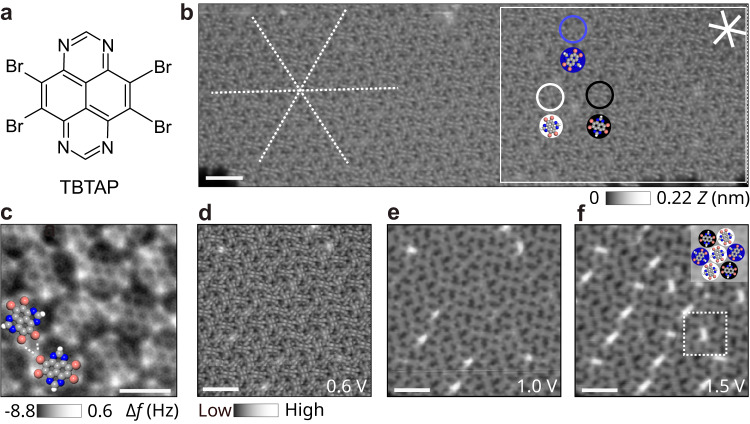
Orientational glassy phase of TBTAP molecules on Ag(111). **a** Chemical structure of the 4,5,9,10-tetrabromo-1,3,6,8-tetraazapyrene (TBTAP) molecule. **b** STM overview image of the TBTAP molecular domain having an orientational glassy phase. The White, black and blue circles and the corresponding models below the circles show the three molecular orientations in the assembly with respect to the $\langle 10\overline{1} \rangle$ directions of Ag(111) (plain white lines), (*I* = 100 pA, *V*_s_ = 600 mV). The red, blue, gray and white spheres in the molecular model represent bromine, nitrogen, carbon and hydrogen atoms, respectively. The white dotted lines indicate that the molecular assembly forms a hexagonal tiling in relative registry with the substrate. The random orientations of molecules in the white frame (a detailed molecular pattern can be found in Supplementary Fig. 2) show an orientational glassy phase of TBTAP molecules on Ag(111). Scale bar: 2 nm. **c** Constant-height AFM image with a CO-terminated tip resolving the chemical structure of each TBTAP molecules, the assembly is stabilized by halogen bonding (*A*_osc_ = 50 pm). Scale bar: 1 nm. **d–f** Series of STM images acquired at different voltages. As *V* increases, few molecules appear brighter due to the particular configuration of the the six surrounding molecules (see the white dotted frame and inset in **f**). It consists of an uniform distribution of six molecules aligned along the main orientations of the substrate. (*I* = 100 pA). Scale bar: 2 nm.

Herein, we demonstrate that 4,5,9,10-tetrabromo-1,3,6,8-tetraazapyrene (TBTAP) molecules (Fig. 1a) adsorbed directly on an Ag(111) surface can exhibit pronounced vibronic features in d*I*/d*V* spectroscopic measurements. Self-assembly of TBTAP molecules on Ag(111) leads to an orientational glassy phase, as determined by high-resolution STM and atomic force microscopy (AFM). Due to their electron-accepting nature, TBTAP molecules are charged by a single electron donated from the substrate, which is confirmed by a Kondo resonance observed in tunneling spectroscopy. The discharge of TBTAP^•−^ can be locally controlled by the electric field of the tip, leading to conductance resonances at positive voltages in d*I*/d*V* spectra and concentric rings in d*I*/d*V* maps. These peaks result from the discharge of the molecule followed by vibrational excitations in accordance with the FC model. Our work thus provides an alternative route to study *e*–*ν* coupling of molecules self-assembled on a metal substrate through an appropriate design of the precursors.

## Results

### Orientational glassy phase of TBTAP molecules on Ag(111)

TBTAP was prepared following the literature procedure^39,40^. Molecules were sublimated on the Ag(111) surface kept at a temperature of ~100–150 K (see the “Methods” section). Such cold deposition not only prevents the dehalogenation reaction of the molecule^43^ but also leads to their spontaneous assembly into extended molecular islands, as shown in the STM overview of Fig. 1b. In these domains, each TBTAP molecule has a *H* shape contrast in agreement with the molecule’s dimension and is adsorbed along the three main [10$\overline{1}$] directions of Ag(111). These adsorption orientations are denoted by white, black and blue circles, respectively. Using constant-height AFM imaging with CO-terminated tips (Fig. 1c)^44^, we also resolved the chemical structure of individual TBTAP molecule within the assembly. We conclude that the molecules lie flat on Ag(111) and are stabilized by non-covalent Br ⋯   Br and Br ⋯  N halogen bonds (white dashed lines in Fig. 1c) and by C–H ⋯  Br and C–H ⋯  N hydrogen bonds (Supplementary Fig. 1)^41–43^. The white dotted lines of Fig. 1b indicate that the molecular assembly forms a perfect hexagonal tiling in relative registry with the underlying substrate (plain lines). Along each dotted line, molecules are randomly rotated by ≈ ±60° with respect to each other (Supplementary Fig. 2). This is likely induced by the limited rotational dynamics of molecules in the assembly at the low temperature which restricts a rearrangement of the molecular lattice. As a result, the TBTAP lattice adopts an orientational glassy phase^45,46^.

We next acquired a series of STM images at the same area by varying the bias voltage from 0.6 to 1.5 V (Fig. 1d–f). While at *V*_s_ = 0.6 V all molecules show a very similar contrast by STM, some of them appear much brighter above *V*_s_ ≥ 1 V (Fig. 1e). A closer look at these bright molecules reveals a particular bonding environment that consists of six molecules with three distinct adsorption orientations. These surrounding molecules have the arrangement as depicted in the inset of Fig. 1f.

### Electronic and magnetic properties of TBTAP molecules

**Fig. 2:**
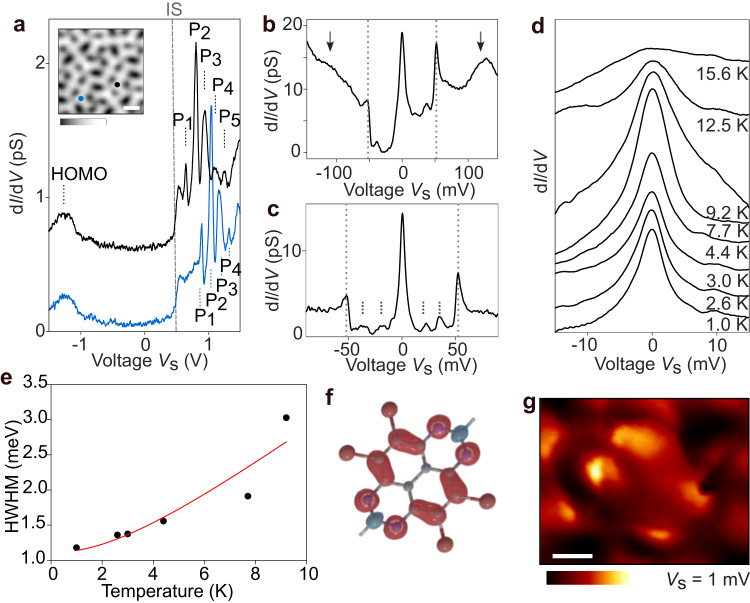
Electronic signature of TBTAP^•−^ molecules in d*I*/d*V* spectroscopy. **a** Exemplary d*I*/d*V* point-spectra (black and blue) acquired at TBTAP molecules (marked by black and blue dots in the inset) of the assembly. The spectra are shifted for clarity. The step feature at 0.5 V is attributed to a hybrid metal-organic interface state (IS) while the resonance at −1.1 V to the HOMO. The *P*_*n*_ peaks are related to the discharging event of the TBTAP^•−^ molecule followed by vibrational excitations (*V*_s_ = 1.5 V; *I*_t_ = 0.5 nA, ${A}_{{{{{{{{\rm{mod}}}}}}}}}$ = 5 mV, *f* = 531 Hz). Scale bar in the inset: 1 nm. **b** d*I*/d*V* spectra near *E*_F_ acquired at the periphery of the molecule, showing a zero-bias peak (ZBP) and intense vibronic side peaks at ±52 mV (gray dotted lines). The broad resonances at about ±110 mV (arrows) are associated to the SOMO/SUMO levels, respectively. **c** Close-up d*I*/d*V* spectra acquired at the center of the molecule. Additional vibronic side peaks are visible at ±18, ±36 and ±52 mV, respectively. A linear background has been subtracted, (${A}_{{{{{{{{\rm{mod}}}}}}}}}$ = 1 mV, *f* = 531 Hz). **d** Temperature dependency of the ZBP from *T* = 1.0; K to *T* = 15.6 K. The ZBP fully vanishes at *T* = 15.6 K. **e** Half width at half maximum (HWHM) of the ZBP peaks extracted from Frota fits and plotted versus *T*. *T*_K_ is estimated to be 8.8 ± 1.5 K. **f** Spin density distribution of TBTAP^•−^ in gas phase. Red (blue) isosurfaces refer to spin up (down) density. **g** Constant-height d*I*/d*V* map of a TBTAP^•−^ molecule at 1 mV (${A}_{{{{{{{{\rm{mod}}}}}}}}}$ = 0.3 mV, *f*  = 531 Hz). Scale bar: 200 pm.

We next investigate the electronic properties of TBTAP molecules using differential conductance measurements. Figure 2a shows exemplary d*I*/d*V* point-spectra acquired at TBTAP molecules (blue and black dots in the STM inset). A step-like feature at *V*_s_ ≈ +0.5 V (gray dashed line) is systematically observed which is attributed to an interfacial state (IS) similar to those observed for epitaxial PTCDA or NTCDA molecules on Ag(111)^47–49^. At higher voltages, a series of intense resonance peaks denoted *P*_*n*_ (*n* is an integer) appear starting from *P*_1_ = 0.6 V (0.9 V) for the blue (black) spectra. A slight shift of the *P*_1_ energy is often observed between neighboring molecules of the assembly, as exemplary shown in Supplementary Figs. 3 and 4. We also assign the resonance at *V*_s_ = −1.1 V to the highest occupied molecular orbital (HOMO, Supplementary Fig. 5). As will be discussed in the following, we attribute the sequence of *P*_*n*_ peaks to two concomitant mechanisms: first the charge-state transition from the radical anionic species TBTAP^•−^ molecule to its neutral counterpart TBTAP^0^ induced by the electric field of the tip^30–32^ and second, the associated vibrational excitations of the molecule^50^.

Upon adsorption to Ag(111), the lowest unoccupied molecular orbital (LUMO) of the neutral TBTAP shifts below *E*_F_ and becomes populated by a single electron leading to a singly occupied molecular orbital (SOMO) of spin 1/2 (Supplementary Fig. 5a). Due to Coulomb repulsion, the SOMO level is accompanied by the formation of a resonance above *E*_F_ (i.e. singly unoccupied molecular orbitals—SUMO) separated by the Coulomb energy *U* as reported by Fernandez-Torrente et al. in the case of TCNQ^−^ molecules on Au(111)^51^. To confirm the radical anionic character of the TBTAP molecule on Ag(111), we then focus using d*I*/d*V* spectroscopy on energies close to the Fermi level.

Figure 2b shows the zero-bias peak (ZBP) detected in d*I*/d*V* spectra at the center of the molecule, which is always accompanied by two pronounced side peaks at ±52 mV (gray dotted lines). The resonances at ±110 mV (black arrows) are assigned to the SOMO/SUMO states of the molecule. As compared to the ZBP, they are much broader, symmetric to *E*_F_ and separated by *U* ≈ 220 mV. Note that a variation of their position in energy was estimated between 90 and 110 meV among molecules of the glassy phase (Supplementary Fig. 5b). Using d*I*/d*V* mapping, we also confirmed that the SUMO and SOMO orbitals are identical as a result of their splitting in the anionic TBTAP^•−^ (Supplementary Fig. 5c). Figure 2c displays a d*I*/d*V* spectra acquired at the periphery of the molecule, where the ZBP becomes more pronounced as compared to the side peaks. In analogy to ref. ^51^, we assign the ZBP to the manifestation of a Kondo singlet from the TBTAP^•−^ electron trapped in the SOMO level while side-peaks are related to inelastic vibrational excitations of the molecule. Two more pairs of resonances are observed at ±18 and ±36 mV with much lower intensity (dashed lines in Fig. 2c). We think that they might be also related to low-vibronic modes of the TBTAP molecule.

To confirm the Kondo nature of the ZBP, d*I*/d*V* spectra were acquired at varying sample temperatures *T*. Figure 2d shows a significant broadening of the ZBP with increasing *T*, which fully vanishes for *T* = 15.6 K. For each spectrum, we extracted the half width at half maximum (HWHM) and plotted it as a function of the temperature *T* (Fig. 2e). A typical Kondo-screened state is deduced with an estimated Kondo temperature of *T*_K_ ≈ 8.8 ± 1.5 K using the Fermi liquid model^52^. Using density functional theory (DFT), we also calculated the spin density of the TBTAP^•−^ in gas phase (Fig. 2f). Its distribution is mostly located at the periphery of the TAP core, in relative agreement with the localization of the ZBP over the TBTAP^•−^ molecule obtained by zero-bias d*I*/d*V* mapping (Fig. 2g). From these spectroscopic data, we infer the ground-state of the radical TBTAP^•−^ molecules on Ag(111) as a result of the occupancy by a single electron of its former LUMO orbital. This “charged” TBTAP configuration can be pictured as the analog of a singly-occupied quantum dot, whose charge-state is modified by the local field of the STM tip.

### Experimental signature of electron–vibration coupling

**Fig. 3:**
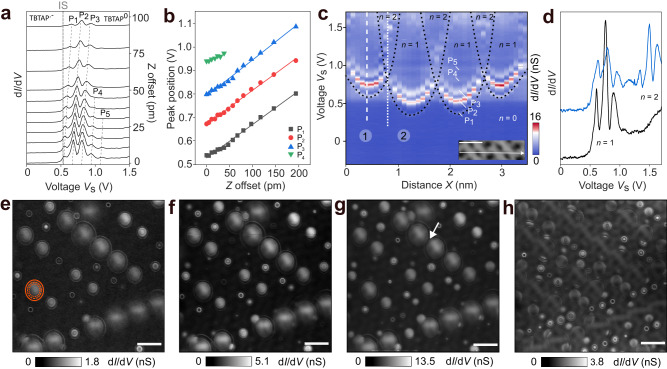
Contribution of the TBTAP vibrational excitations to the Coulomb pattern. **a** Series of d*I*/d*V* point-spectra above a TBTAP^•−^ molecule with increasing tip-sample separation *Z*, (${A}_{{{{{{{{\rm{mod}}}}}}}}}$ = 5 mV, *f* = 531 Hz). At close tip–sample separation, up to five resonance peaks (*P*_1_–*P*_5_) can be observed. **b** Peak positions observed in **a** as a function of the *Z* offset. Black squares, red circles, blue up-pointing triangles and green down-pointing triangles correspond to the *P*_1_, *P*_2_, *P*_3_ and *P*_4_ peaks in **a**, respectively. **c** d*I*/d*V*(*V*,*X*) cross-section acquired along four molecules (white arrow in the inset). The hyperbola marked by black dotted contour lines and centered to the TBTAP^•−^ molecules refers to the discharge event (*P*_1_ peak). They represent the threshold voltage at which one electron is removed per molecule (area *n* = 1). Scale bar in the inset: 1 nm. **d** d*I*/d*V* spectra were acquired near the white dotted line of **c** across molecule 1 and 2. **e–g** Constant-height d*I*/d*V* maps showing the evolution of Coulomb rings at constant *V*_s_ = 0.5 V upon approaching the tip by *Z* = 50 pm between each map. The gating efficiency is increased with decreasing *Z* leading to the appearance of more discharging rings. Each occurrence is composed of three concentric rings (depicted by red rings) centered to the TBTAP^•−^ molecules indicating that vibrational excitations are associated with the discharge event of the anionic state. Adjacent rings of neighboring molecules intersect at high voltage, as indicated by the white arrow in **g** (*A*_osc_ = 10 mV, *f* = 531 Hz). Scale bar: 2 nm. **h** The constant-height d*I*/d*V* map at *V*_s_ = 1 V. The concentric rings are still present but are hindered by the contribution IS state to the d*I*/d*V* maps. Scale bar: 2 nm.

We next investigate the influence of the tip-sample distance *Z* on the discharging/vibronic *P*_*n*_ peaks above the IS onset (gray dotted line) by recording a series of d*I*/d*V* point-spectra with increasing *Z* (Fig. 3a). The first peak *P*_1_ is assigned to the threshold voltage at which the discharge of the TBTAP^•−^ molecule occurs by tip gating. Such tip-assisted discharge of a molecular radical at surfaces is usually described using a double barrier tunneling junction (DBTJ)^30–32^. Within this model, the lever arm *α* is a key parameter that reflects the efficiency with which the STM can locally gate the molecular level and provoke its discharge. Considering a plate-capacitor geometry, this voltage drop *α* between molecule and substrate linearly depends on the (*X*, *Y*, *Z*) tip positions with respect to the molecule as well as the voltage *V*_s_ applied to the substrate. As described in ref. ^30^, *α* can be estimated by the ratio *E*_SOMO_/(*E*^−→0^−*E*_SOMO_) with *E*_SOMO_ is the SOMO energy and *E*^−→0^ = *e* ⋅ *P*_1_ is the discharging energy. In our experiments, *P*_1_ is found to vary from 0.35 up to 0.8 V within a molecular assembly using the same tip conditions and tip–sample separations (Fig. 1a, see also Supplementary Fig. 3) whereas the SOMO energy is ≈ − 90–110 meV between TBTAP^•−^ molecules (Fig. 2b, Supplementary Fig. 5). Thus, we estimate the voltage drop in our system to be in the range of *α* = 0.12–0.35. This value is in a similar range compared to previous works of molecules on metal^30^.

Increasing the tip–sample distance *Z* between d*I*/d*V* spectra (Fig. 3a) systematically leads to the shift of the *P*_*n*_ peaks towards higher voltage values (dashed lines) since the fraction *α* decreases with the tip retraction. All peaks decrease in intensity as *Z* increases or even vanish (*P*_4_ and *P*_5_ for *Z* ≥ 50 pm) due to the small tunneling current in the STM junction at such tip–sample separation^27^. Figure 3b shows the *P*_*n*_ peak positions extracted from Fig. 3a as a function of *Z* offset, where each collection of data-points is fitted with linear functions. Their slopes *a* slightly increase with *n* such as ${a}_{{{{{{{{\rm{{P}}}}}}}_{{{{{{{{\rm{1}}}}}}}}}}}}$ = 1.425 mV pm^−1^, ${a}_{{{{{{{{\rm{{P}}}}}}}_{{{{{{{{\rm{2}}}}}}}}}}}}$ = 1.438 mV pm^−1^ and ${a}_{{{{{{{{\rm{{P}}}}}}}_{{{{{{{{\rm{3}}}}}}}}}}}}$ = 1.544 mV pm^−1^, which is in analogy to ref. ^27^. The fit lines are also shifted in voltage by Δ*V* ≈ 120–150 mV extracted from several experimental data-sets. More precisely, the spacing between *P*_1_ and *P*_2_ is always about 10% larger than between *P*_2_ and *P*_3_ and 10% shorter than the *P*_3_/*P*_4_ spacing. If the molecular degrees of freedom are considered within the harmonic approximation, the energy difference between vibrationally excited states of a single mode should be equal leading to equally-spaced resonance peaks. However, this may not be a realistic representation of the STM setup where anharmonic effects may result in peaks that are not evenly spaced. Additionally, the tunneling of current through the STM device induces a charge redistribution that results in an atomic rearrangement^53^, thereby altering the vibrational spectrum.

Assuming that the vibrational excitation peaks correspond to a single vibronic mode of the molecule, we estimate the vibration energy ${{\Omega }}_{\exp }$ taking into account the voltage drop *α* such as ${{\Omega }}_{\exp }$ = *α*Δ*V*. With the large variation of *α* = 0.15–0.35 between neighboring molecules, the absolute value of ${{\Omega }}_{\exp }$ ranges from 14 up to 53 meV, which is in the range of the values obtained by tunneling through the Kondo resonance (Fig. 2c). Since *α* remains constant at one molecule site, the error on the estimate of single mode energy ${{\Omega }}_{\exp }$ is about ± 10% (i.e. maximum 5 meV) as it scales with Δ*V*.

We last compare this experimental value to the infrared spectrum (FT-IR) of TBTAP in solution (Supplementary Fig. 6). Although the FT-IR spectrum of polycyclic aromatic molecules is sensitive to their charge states whereby these modes are activated by ionization, the frequency shifts are limited to about 100 cm^−1^ (i.e. 12 meV) relative to the neutral state^54^. As compared to Supplementary Fig. 6, the energy range of ${{\Omega }}_{\exp }$ corresponds to the vibrational mode of the peripheral C–Br bonds (peak 4 at 79 ± 12 meV) as well as vibrational-rotational of the pyrene structure (peak 1 at 50 ± 12 meV). Note also that these values corresponding to the single molecule in gas phase are likely overestimated as compared to the case of TBTAP coupled to the Ag(111) surface.

Figure 3c shows a d*I*/d*V*(V, X) cross-section acquired along four neighboring molecules (white arrow of the STM inset). The hyperbola of high conductance marked by black dotted contour lines correspond to the discharge event *P*_1_ detected above the molecules as a function of the lateral tip position *X*. The contour lines thus pinpoint the charge-state transition from radical anions TBTAP^•−^ molecules to neutral TBTAP^0^ as a function of the tip position *X* and the applied voltage *V*_s_^29–32^. Each hyperbola is centered to the molecular sites indicating the localization of the charge before its removal by gating. For voltages *V*_s_ greater than values of the contour lines (area *n* = 1), one electron is successfully removed from one molecule. For increasing *V*_s_ above a molecule (white dashed line in Fig. 3c), two more hyperbola of high intensities (denoted *P*_2_ and *P*_3_) emerge which are followed by a fainter oscillation, which maxima are marked *P*_4_ and *P*_5_. These features correspond to the sequence of discharging/vibrational excitation peaks exemplary shown in Fig. 3a. These resonances are systematically observed above each molecule but can be slightly shifted in energy as a result of a modulation of the surface potential within the molecular assembly.

As expected for the DBTJ model^27–32^, increasing *V*_s_ leads to the expansion of the branches of each discharge hyperbola up to their crossing. Such crossing point, whose position in *X* is marked by a white dotted line in Fig. 3c, corresponds to the equidistance of the tip from the neighboring molecules 1 and 2. At voltage values corresponding to the area *n* = 2, the removal of two electrons by gating (one from each molecule) becomes possible, which indicates that two neighboring molecules can be subsequently excited at this tip position. To illustrate this, Fig. 3d compares d*I*/d*V* spectra of Fig. 3c extracted along the white dashed lines (molecule 1) and white dotted lines (between molecules 1 and 2), respectively. Both curves show the typical series of discharging/vibronic oscillations *P*_*n*_ at voltages between 0.6 and 1.0 V corresponds to region *n* = 1. At higher voltage (*V*_s_ ≥ 1.2 V), no more resonances are observed when placing the tip on the molecule whereas a second series of oscillations emerges when probing between the two molecules (*n* = 2 in blue spectra). The resurgence of the oscillations at the region *n* = 2 of Fig. 3c (see also Supplementary Fig. 3) suggests the vibrational excitations of both neighboring molecules at such tip position. This can be explained by a slight difference of the SOMO levels between these neighboring molecules or a local variation of their electrostatic environment.

Charging/discharging events triggered by tip gating in the case of 0D-quantum dot systems appear in d*I*/d*V* mapping at a given *V*_s_ as rings of high differential conductance also called “Coulomb” rings^29–32^. To explore the contribution of the vibronic modes to the Coulomb pattern, we acquired a series of constant-height d*I*/d*V* maps at constant voltage *V*_s_ = 0.5 V while approaching the tip by 50 pm between maps (Fig. 3e–g). The d*I*/d*V* maps show three concentric rings centered to anionic TBTAP^•−^ molecules indicative of their succesfull discharge and the subsequent vibrational excitations (molecules are turned into the neutral TBTAP^0^ state inside the ring)^29^. The Coulomb ring with the largest diameter always corresponds to *P*_1_ while the internal ones are associated to the vibronic modes *P*_2,3,.._in agreement with the d*I*/d*V*(X, V) cross-section of Fig. 3c. Note that as *Z* decreases for a constant-voltage, the electric field of the tip is more efficient to discharge molecules resulting in the appearance of more rings over the assembly.

The d*I*/d*V* map at *V*_s_ = 1.0 V (Fig. 3h) shows even more ring features centered to TBTAP molecules as compared to Fig. 3e–g. However, their observation is more tedious since the contrast is hampered by the strong contribution of the IS state above *V*_s_ = 0.5 V to the d*I*/d*V* maps. We also emphasize that the variable emergence of the Coulomb rings is the result of the complex electrostatic potential experienced by each molecule in the orientational glassy phase due to the neighboring ones. Adjacent rings arising from neighboring molecules intersect at high voltage (white arrow in Fig. 3g) but do not fuse implying the absence of electron correlations between molecules during the discharging process^55^.

## Discussion

**Fig. 4:**
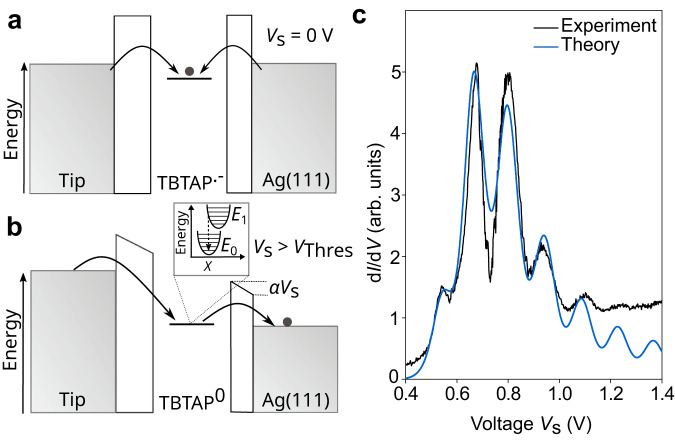
Models and simulations of electron–vibration coupling in TBTAP. **a** and **b** Schematic representation of the transport processes. At equilibrium (*V*_S_ = 0 V), the anionic TBTAP^•−^ molecule is occupied by one electron transferred from the substrate (**a**). At the threshold bias voltage, the SOMO level aligns with the chemical potential of the surface allowing a resonant transport processes and the vibrational excitations of the molecules via the Franck–Condon model (**b**). **c** d*I*/d*V* measurement (black) and simulated curve (blue) by the Born–Markov master equation method.

We interpret the peaks in Fig. 3a as the tip-induced discharging of the TBTAP^•−^ radical in the form of a double barrier tunneling junction model in combination with a FC model. At low bias voltages, a single electron is trapped on each TBTAP molecule (Fig. 4a). When the threshold voltage ${V}_{{{{{{{{\rm{Thres}}}}}}}}}$ is reached, the electrons can tunnel from the molecule towards the substrate (Fig. 4b), leaving the molecule in a neutral state above ${V}_{{{{{{{{\rm{Thres}}}}}}}}}$. As a result of the discharging process, the current exhibits the characteristic step-like increase for the FC model, causing sharp peaks in the conductance. The molecular vibrations are strongly coupled to the charge transport, resulting in the onset of a FC blockade. Note also that the detection of such discharging/vibronic events of the TBTAP^•−^ is not restricted to tunneling measurements since preliminary force spectroscopic data (Supplementary Fig. 7) have also detected features similar to those obtained by AFM using single redox molecules^56^. Future AFM-based investigations will address this in more details.

To support the interpretation of the experimental results as a strong coupling between electronic and vibrational degrees of freedom, we theoretically investigate the charge transport in a system that corresponds to an approximation of the experimental setup. As discussed above, we model the STM setup using a DBTJ with a single vibrational mode and a single electronic state corresponding to the SOMO. We investigate this model by employing a Born–Markov master equation approach^3,57,58^, which treats the setup within second-order in the environment–molecule coupling. The parameters defining the model are determined by fitting the conductance computed by the transport method to the experimentally measured curves depicted in Fig. 3a for *Z* = 0. For additional information regarding the utilized model system and transport formalism, we refer to Supplementary Note 1.

Relevant parameters for the interpretation include, for example, the coupling of the molecule to the tip and substrate, which have been determined to Γ_T_ = 100 neV and Γ_S_ = 580 neV, respectively. It is noted that within the Born–Markov master equation used, these coupling parameters determine the overall strength of the current but not the width of the resonance peaks. The lever arm associated with the asymmetric coupling is *α* ≈ 0.15, which lies within the range of the experimental values. Our numerical fitting procedure estimates the vibrational frequency to Ω_Theory_ = 20 meV, and a dimensionless electronic-vibrational coupling strength to *λ*/Ω = 2.4, corresponding to a Huang–Rhys factor^59^ of *R* ≈ 3.4. Figure 4c depicts the fitted conductance–voltage characteristics employing the previously described parameters. The convincing agreement between the model and the experiments justifies the use of a single-state single-mode model system.

The weak coupling between molecules and the environment is crucial for the strong signature of electronic–vibrational coupling to emerge. Four Br atoms in each molecule could be the key aspect to lift the molecular backbone from the substrate. In order to examine this argument, debromination of TBTAP were performed by annealing the sample to ≈400 K. Supplementary Fig. 8 shows the honeycomb metal-organic frameworks formed by (MOFs)–TBTAP–Ag_2_. As reported recently^43^, organometallic C–Ag–C bonds are the main building blocks for the metal-organic frameworks. Within this framework, there are no longer any electron–vibration features in the d*I*/d*V*-spectra. The dehalogenation process strongly enhances the coupling between molecules and substrates, and forms a relatively strong C–Ag–C bond between molecules and substrates.

To further investigate the radical state of TBTAP molecules on Ag(111), we employed the STM tip to relocate the molecules from the island to the exposed surface. Supplementary Fig. 9a and b depict our successful transfer of a TBTAP molecule from the molecular island to the clean surface. However, obtaining spectra from the isolated molecule poses significant challenges due to its high mobility induced by the STM tip on the free surface. Despite this, we managed to construct dimers successfully, which revealed the presence of Coulomb rings in both molecules (supplementary Fig. 9c and d). This observation provides compelling evidence that TBTAP molecules exhibit radical-charged characteristics upon absorption on the Ag(111) surface.

In summary, we employed high-resolution STM and AFM images to observe a pattern of gate-dependent vibrational excitations in the orientational glassy phase of self-assembled TBTAP molecules on Ag(111). The TBTAP molecules are in a negatively charged radical state due to the electron donation of the substrate, as evidenced by the observation of a Kondo resonance in STS. d*I*/d*V* spectra further demonstrate the capability of discharging the molecule by tip gating and a strong electron–vibration coupling in molecules. Further dehalogenation experiments have proven that the weak interactions between molecules and substrates are due to the presence of the four Br atoms. Model calculations confirm the interpretation of the experimental data. Our results provide a feasible way to study electron–vibration couplings in molecules on the metal surface through a chemical design of molecular structures.

## Methods

### Sample preparation

The Ag(111) surface (MaTeck GmbH, 99.999%) was cleaned by cycles of Ar ion sputtering and annealing. TBTAP molecules were sublimated at ≈440 K with the substrate kept at ≈100–150 K.

### STM/STS experiments

STM and d*I*/d*V* experiments were performed with a low-temperature (1 K) Joule–Thomson STM/AFM microscope (purchased from Omicron GmbH) in ultra high vacuum (UHV) of ≈ 10^−10^ mbar operated with a Nanonis RC5e electronics. Differential conductance, d*I*/d*V*, spectra were recorded with a lock-in amplifier using modulation amplitudes indicated in the caption. The d*I*/d*V*(*X*,*V*) of Fig. 3d consists of 40d*I*/d*V* spectra of 512 pixels each separated by ≈90 pm along the *X* direction (i.e. 3.2 nm × 2.2 V for 40 × 512 pixels^2^). Raw d*I*/d*V* spectra are shown in a waterfall plot (Supplementary Fig. 3).

### AFM experiments

AFM experiments were performed in another low-temperature microscope (4.5 K) equipped with a qPlus sensor (*f*_0_ = 23.7 kHz, *k* = 1800 N m^−1^).^56^ In order to enhance the AFM resolution, the tip was functionalized with a CO molecule. CO functionalizing was done by depositing CO molecules on the cold surface (≤15 K) and then gently indenting the tip on top of a single CO molecule.

### DFT calculations

Gas-phase DFT calculations were performed with the Gaussian 16 package^60^, using the B3LYP exchange-correlation functional^61^ and a 6-31G^⋆⋆^ basis set. To simulate the molecular anion, an extra electron was added and a doublet multiplicity initialized.

### Reporting summary

Further information on research design is available in the Nature Portfolio Reporting Summary linked to this article.

### Supplementary information

Supplementary Information

Reporting Summary

## Data Availability

The data that support the findings of this study are available from the corresponding authors upon request.
