# Supplementary material for: Strong signature of electron-vibration coupling in molecules on Ag(111) triggered by tip-gated discharging
Source: Nat Commun. 2023 Sep 25;14:5956. doi: 10.1038/s41467-023-41601-2 (PMC10519934; doi:10.1038/s41467-023-41601-2)
Supplement: Supplementary file 1 — Supplementary Information [file 41467_2023_41601_MOESM1_ESM.pdf]

Supplementary information for: Strong signature of electron-vibration coupling in molecules on Ag(111) triggered by tip-gated discharging

Chao Li,[*,†,§] Christoph Kaspar,[‡,§] Ping Zhou,[¶,§] Jung-Ching Liu,[†] Outhmane Chahib,[†] Thilo Glatzel,[†] Robert Häner,[¶] Ulrich Aschauer,[¶] Silvio Decurtins,[¶] Shi-Xia Liu,[*,¶] Michael Thoss,[‡,‖] Ernst Meyer,[*,†] and Rémy Pawlak[*,†]

†*Department of Physics, University of Basel, Klingelbergstrasse 82, 4056 Basel, Switzerland*

‡*Institute of Physics, University of Freiburg, Hermann-Herder-Strasse 3, 79104 Freiburg, Germany*

¶*Department of Chemistry, Biochemistry and Pharmaceutical Sciences, University of Bern, Freiestrasse 3, 3012 Bern, Switzerland*

§*equally contributed authors*

‖*EUCOR Centre for Quantum Science and Quantum Computing, University of Freiburg, Hermann-Herder-Str. 3, 79104 Freiburg, Germany*

E-mail: chao.li@unibas.ch; shi-xia.liu@unibe.ch; ernst.meyer@unibas.ch; remy.pawlak@unibas.ch

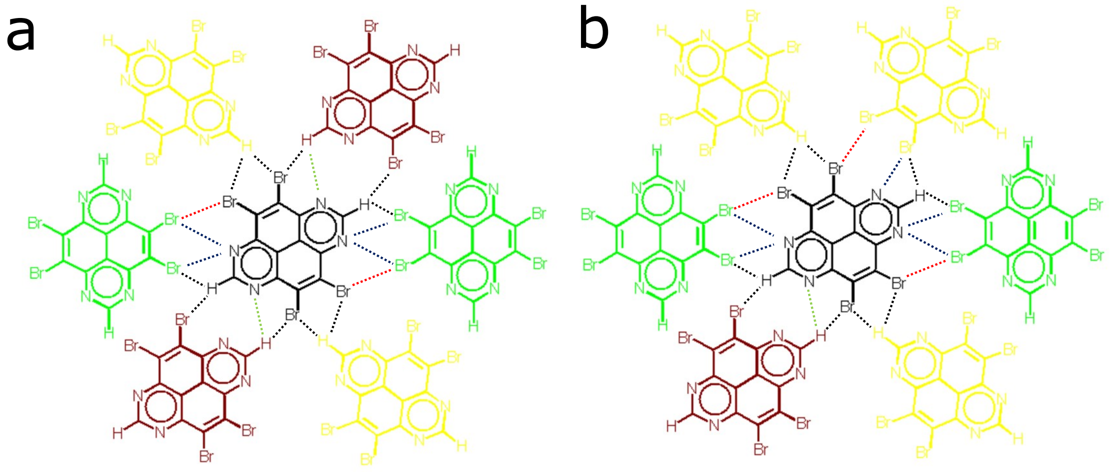

Supplementary Figure 1: Illustration of intermolecular interactions in a small cluster. The local electrostatic environment of center molecule (black) are influenced by the Br$\cdots$Br (red), Br$\cdots$H (black), Br$\cdots$N (blue) and N$\cdots$H (green) interactions. (a) The molecule in the center (black) are surrounded by six molecules with three different orientations in the equivalent positions. (b) Electrostatic environments at the centre of a molecule change when its adjacent molecules change.

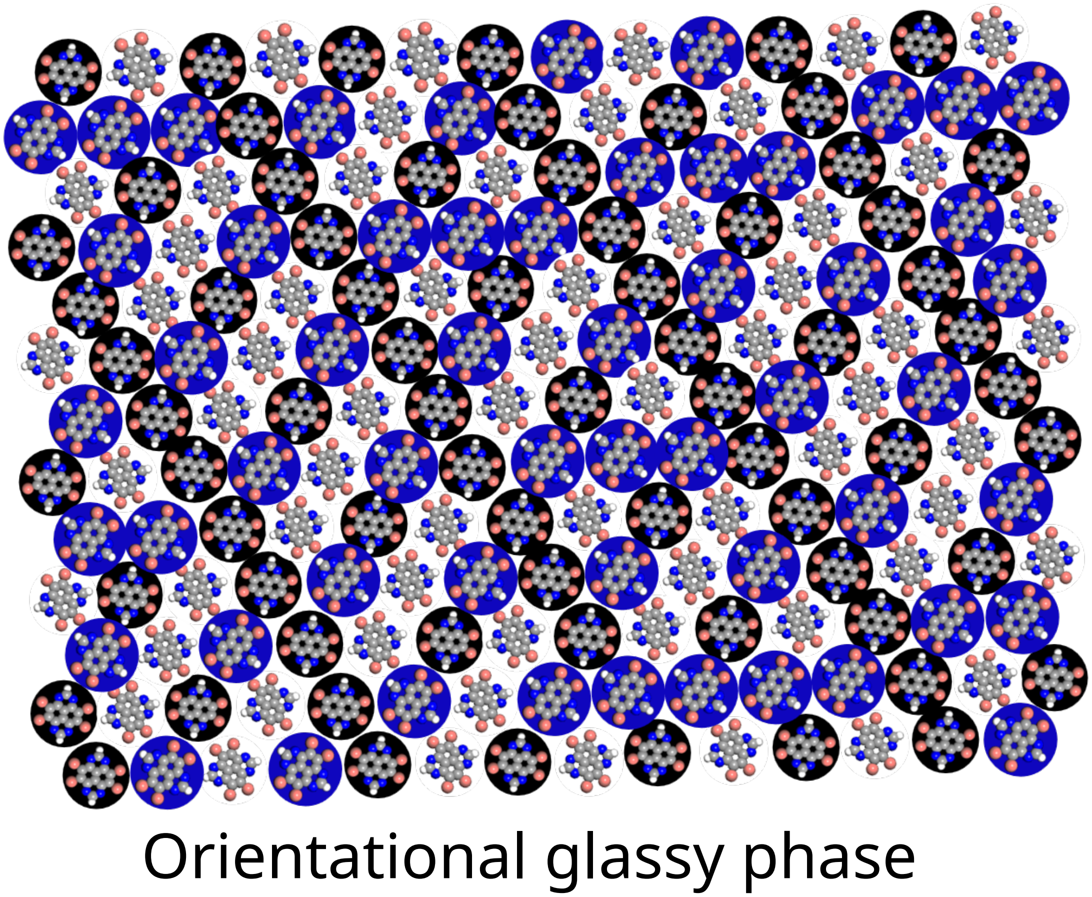

Supplementary Figure 2: The orientational glassy phase of a molecule pattern is made up of three different molecular models. The pattern is a result of molecules in the white dotted rectangle in Figure 1a.

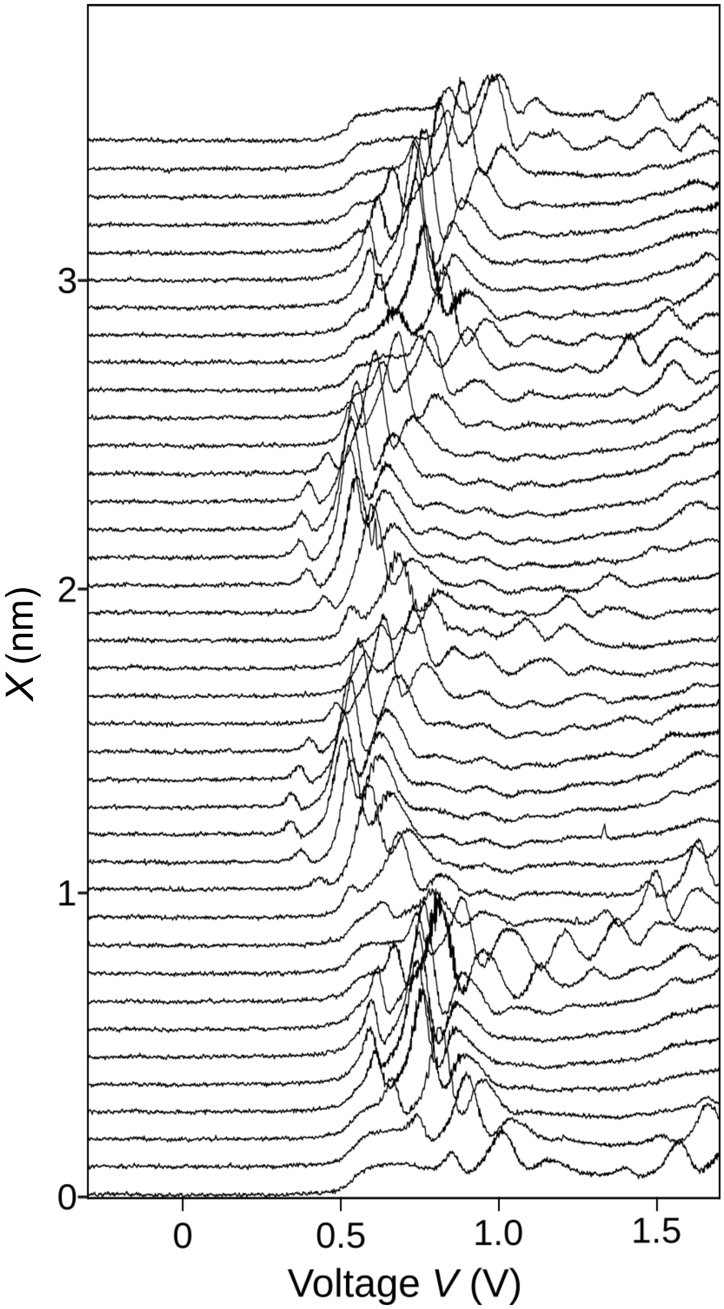

Supplementary Figure 3: Waterfall plot of the dI/dV cross-section shown in Figure 3d.

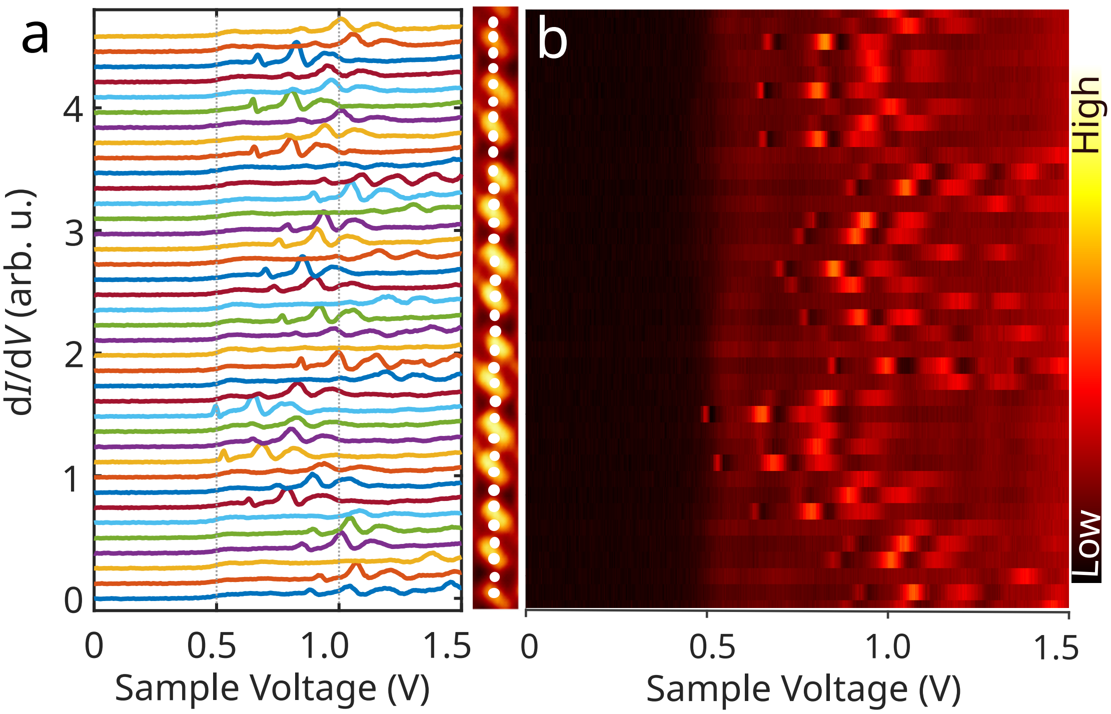

Supplementary Figure 4: (a) Spectra are measured along the fifteen molecules marked by white dotted line. (b) The two-dimensional map of dI/dV line profiles in (a). There is a range of 0.5 V to 1.5 V in the threshold discharging voltages for the fifteen molecules. ($V_{\rm rms} = 10\,\rm mV$).

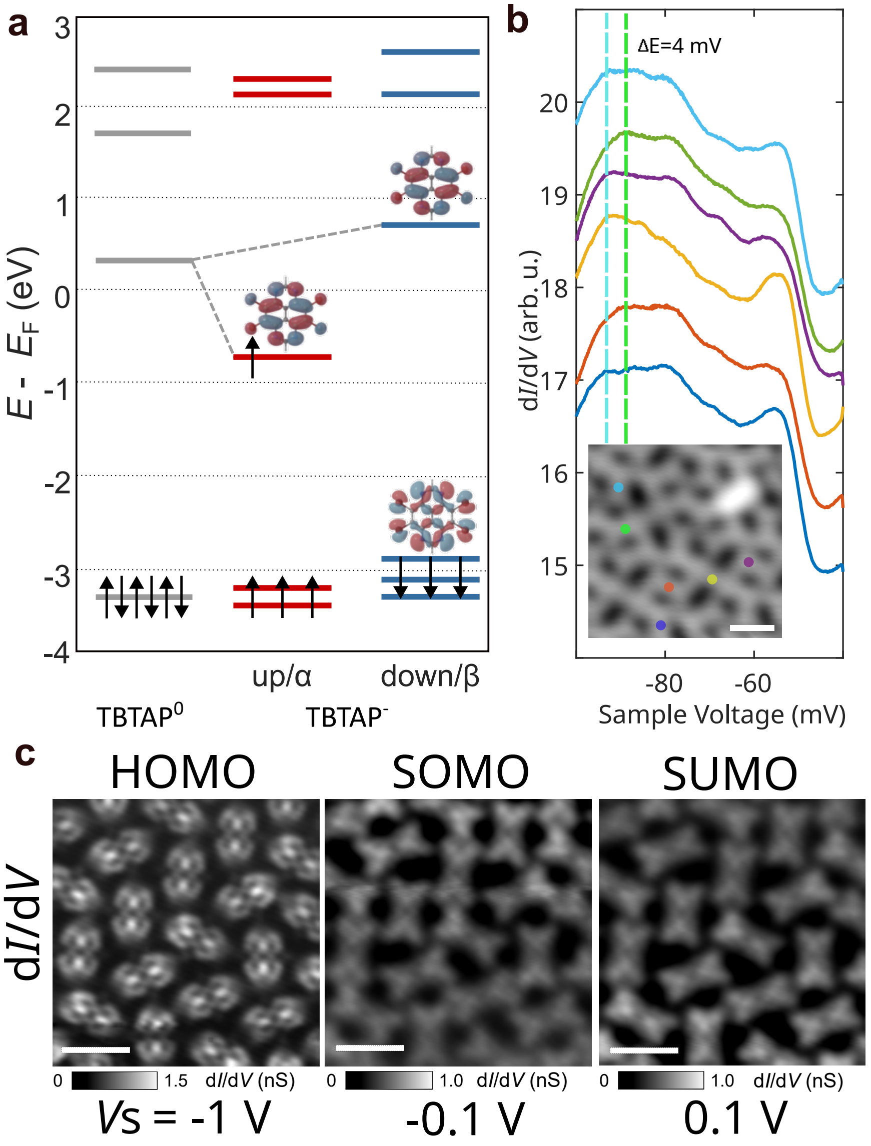

Supplementary Figure 5: (a) a comparison of the nearest-neighbor tight-binding (TB) energy spectrum for the neutral TBTAP^0 (gray) and the charged $\text{TBTAP}^{\bullet -}$ (red and blue). Upon charging, the LUMO splits into SOMO and SUMO. The gas phase DFT calculations yielded molecular orbitals of SOMO, SUMO, and HOMO, which are listed alongside their corresponding correlated orbitals. (b) The $\mathrm{d}I/\mathrm{d}V$ spectra collected from various molecules exhibit a slight dispersion in the SOMO energy levels ranging from 90 to 100 meV, $V_{\text{rms}} =$ 2 mV. (The inset, $I =$ 100 pA, $V_{\text{s}} =$ -100 mV.). (c) Constant height $\mathrm{d}I/\mathrm{d}V$ maps of the TBTAP molecules at 0.1 V (modulation voltage of 2 mV), -0.1 V (modulation voltage of 2 mV) and -1 V (modulation voltage of 8 mV), respectively. Scale bars: 1 nm.

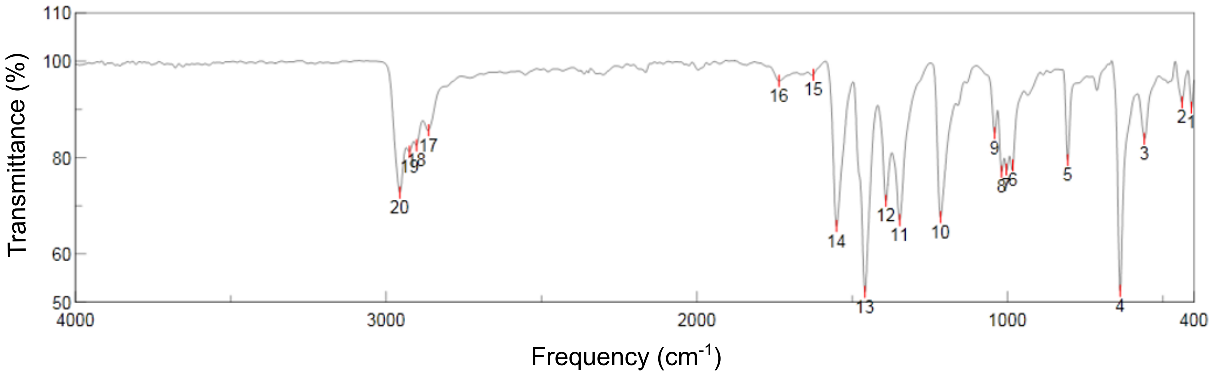

Supplementary Figure 6: FT-IR spectrum of TBTAP. The range of 1200-1550 cm^{-1} (0.15 eV - 0.19 eV), reflects the C-C and C-N skeletal vibrations.

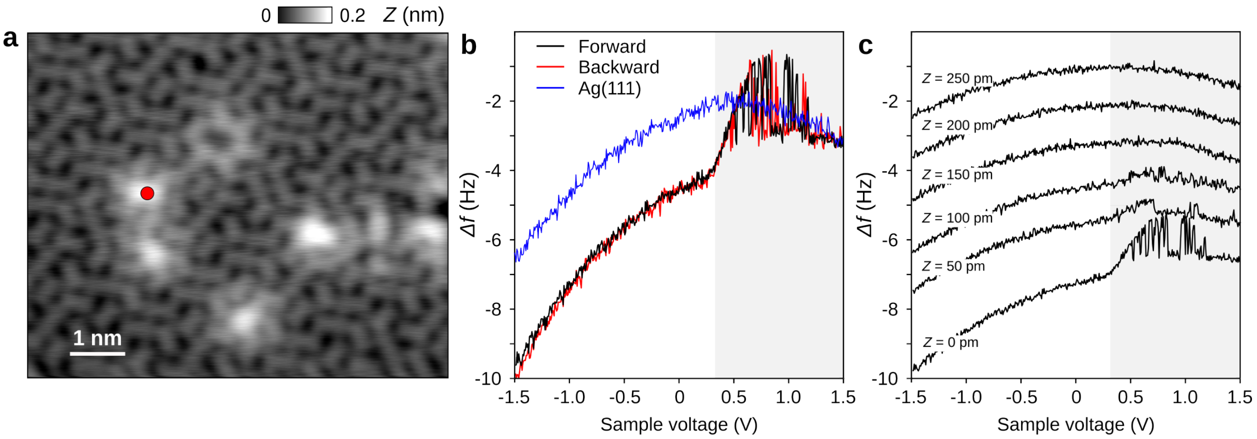

Supplementary Figure 7: Force spectroscopy above the TBTAP molecule marked by a red dot (a) shows the vibration excitation from a threshold sample voltage of 0.6 V (b and c).

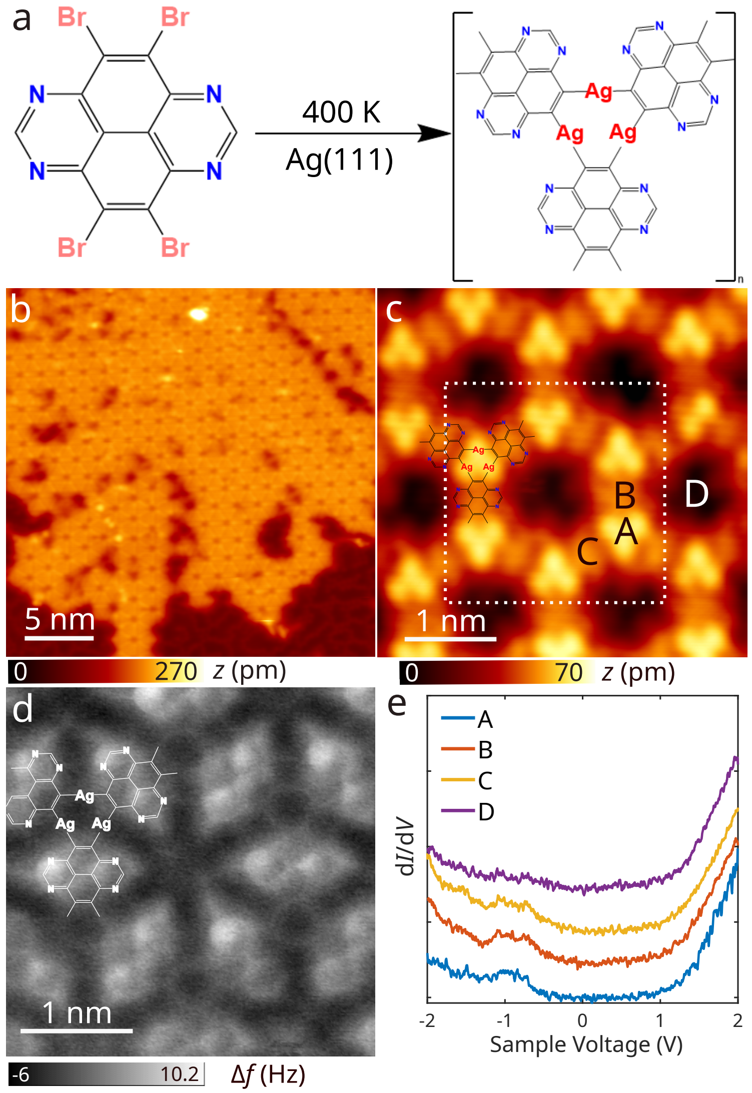

Supplementary Figure 8: Formation of honeycomb metal-organic frameworks by the dehalogenation of TBTAP molecules on Ag(111). (a) Chemical structures of TBTAP and final products and the process of the reaction. (b) Formation of metal-organic frameworks after annealing the sample to ≈ 400 K ($I = 40$ pA, $V_s = 2$ V). (c) Enlarged STM image of the MOFs. The chemical structure of the final product is overlaid on its related molecules ($I = 40$ pA, $V_s =$ -500 mV). (d) Constant height Δf image of MOFs recorded with a CO tip. (e) STS ($V_{rms} = 10\,\mathrm{mV}$) taken on A, B, C and D positions marked in (c).

Supplementary Table 1: Characteristics of the peaks in Supplementary Fig. 6

Peak number	Position (cm^{-1})	Transmittance (%)
1	407.871	90
2	438.726	91
3	559.255	84
4	637.358	52
5	806.099	79
6	982.554	78
7	1003.77	77
8	1019.19	77
9	1041.37	85
10	1215.9	68
11	1347.03	67
12	1391.39	71
13	1458.89	52
14	1550.49	66
15	1624.73	97
16	1735.62	96
17	2863.77	86
18	2902.34	83
19	2924.52	81
20	2956.34	73

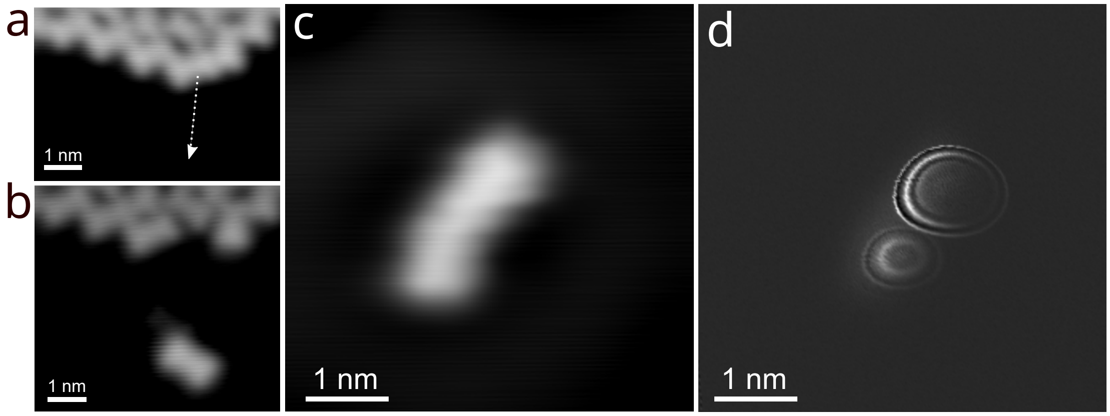

Supplementary Figure 9: (a) Topography of TBTAP island edge (0.3 V, 100 pA). The arrow indicates the trajectory followed during the manipulation of molecules using the STM tip. (b) Same area after manipulations (0.3 V, 50 pA). Isolated TBTAP molecule has a H shape contrast. (c) A dimer constructed by TBTAP molecules (80 mV, 100 pA). (d) Constant height dI/dV map shows the Coulomb rings exist in the dimer at V_s= 1 V.

Supplementary Note 1

To support the interpretation of the experimental results, we approximate the STM setup by employing the following Hamiltonian, which describes a model system with a single electronic state and single vibrational mode [4, 3, 6]:

$$H = \epsilon d^\dagger d + \hbar \Omega a^\dagger a + d^\dagger d \lambda \left(a^\dagger + a\right) + \sum_{k \in \mathrm{S/T}} \epsilon_k c_k^\dagger c_k + \sum_\beta \hbar\omega_\beta b_\beta^\dagger b_\beta$$
$$+ \sum_{k \in \mathrm{S/T}} \left(V_k c_k^\dagger d + V_k^* c_k d^\dagger \right) + \left(a^\dagger + a\right) \sum_\beta \Lambda_\beta \left(b_\beta^\dagger + b_\beta \right). \quad (1)$$

In our simplified model, the operator $d/d^\dagger$ annihilates/creates an electron on the molecule with energy ϵ. The molecular electronic state couples to a vibrational mode, which is described in harmonic approximation with frequency $\hbar\Omega$. The vibrational annihilation/creation operators are denoted with $a/a^\dagger$. Thereby, the parameter λ characterizes the interaction strength between the electronic state and the vibrational mode. Both the substrate (S) and tip (T) are modeled as a continuum of electronic states with energy ϵ_k. The corresponding annihilation/creation operator is denoted by $c_k/c_k^\dagger$. The environmental electronic states couple to the molecular state with the tunneling matrix elements V_k. To account for vibrational relaxation, the mode is additionally coupled to a dissipative environment [2], which we model as a set of harmonic oscillators with frequencies $\hbar\omega_\beta$ and corresponding annihilation and creation operators b_β and $b_\beta^\dagger$.

The influence of the environment on the molecular dynamics is encoded in the spectral density functions

$$\Gamma_{\mathrm{S/T}}(\epsilon) = 2\pi \sum_{k \in \mathrm{S/T}} |V_k|^2 \delta(\epsilon - \epsilon_k), \quad (2)$$

$$J(\omega) = \pi \sum_\beta |\Lambda_\beta|^2 \delta(\omega - \omega_\beta). \quad (3)$$

For simplicity, we describe the substrate and tip within the wide-band approximation, which

implies energy-independent spectral density functions $\mathit{\Gamma}_{\mathrm{S/T}}(\epsilon) \equiv \mathit{\Gamma}_{\mathrm{S/T}}$. For the characterization of the relaxation strength, we define the dimensionless parameter[7]

$$\gamma = \frac{J(\mathit{\Omega})}{\mathit{\Gamma}_{\mathrm{S}} + \mathit{\Gamma}_{\mathrm{T}}}, \tag{4}$$

where $J(\mathit{\Omega})$ is the spectral density of the dissipative environment in Eq. (3), evaluated at the frequency of the mode.

In our simplified model, we assume that the bias voltage drops at the molecule-substrate and molecule-tip contacts. In this context, we define the bias voltage division factor $\alpha \in [0, 1]$, which affects the chemical potentials of substrate μ_{S} and tip μ_{T} by

$$\mu_{\mathrm{S}} = -\,eV\alpha, \tag{5}$$

$$\mu_{\mathrm{T}} = eV(1-\alpha), \tag{6}$$

where e denotes the elementary charge. Such a potential drop can be associated with an asymmetry in the molecule-substrate and molecule-tip coupling,

$$\alpha = \frac{\mathit{\Gamma}_{\mathrm{T}}}{\mathit{\Gamma}_{\mathrm{S}} + \mathit{\Gamma}_{\mathrm{T}}}, \tag{7}$$

which a STM setup may in general exhibit. [12, 11] This quantity is also referred to as the lever arm.

For the treatment of the Hamiltonian defined by Eq. (1), we employ a system-bath partitioning

$$H = H_{\mathrm{S}} + H_{\mathrm{E}} + H_{\mathrm{SE}}. \tag{8}$$

In the above expression, we have introduced three Hamiltonians, with H_{S} representing the system, H_{E} the environment, and H_{SE} the coupling between the environment and the system,

given by

$$H_{\rm S} = \epsilon d^\dagger d + \hbar\Omega a^\dagger a + d^\dagger d \lambda \left(a^\dagger + a\right), \tag{9}$$

$$H_{\rm E} = H_{\rm F} + H_{\rm B} = \sum_{k\in {\rm S/T}} \epsilon_k c_k^\dagger c_k + \sum_\beta \hbar\omega_\beta b_\beta^\dagger b_\beta, \tag{10}$$

$$H_{\rm SE} = \sum_{k\in {\rm S/T}} \left(V_k c_k^\dagger d + V_k^* c_k d^\dagger \right) + \sum_\beta \Lambda_\beta \left(a^\dagger + a\right) \left(b_\beta^\dagger + b_\beta\right). \tag{11}$$

In the limit of vanishing molecule-environment coupling, the Hamiltonian in Eq. (1) can be diagonalized analytically by the small polaron transformation [9]

$$\begin{aligned} \overline{H} = e^{S_1} H e^{S_1} = \overline{\epsilon} d^\dagger d + \hbar\Omega a^\dagger a + \sum_{k\in {\rm S/T}} \epsilon_k c_k^\dagger c_k + \sum_\beta \hbar\omega_\beta b_\beta^\dagger b_\beta + \sum_{k\in {\rm S/T}} \left(V_k X c_k^\dagger d + V_k^* X^\dagger d^\dagger c_k \right) \\ + \sum_\beta \Lambda_\beta \left(a^\dagger + a\right) \left(b_\beta^\dagger + b_\beta\right) - d^\dagger d \sum_\beta \frac{2\lambda\Lambda_\beta}{\hbar\Omega} \left(b_\beta + b_\beta^\dagger\right), \end{aligned} \tag{12}$$

with

$$S_1 = d^\dagger d \frac{\lambda}{\hbar\Omega} \left(a^\dagger - a\right), \tag{13}$$

$$\overline{\epsilon} = \epsilon - \frac{\lambda^2}{\hbar\Omega}, \tag{14}$$

$$X = \exp\left(\frac{\lambda}{\hbar\Omega} \left(a - a^\dagger\right) \right). \tag{15}$$

Overall, we obtain a Hamiltonian that is diagonal in the reduced system subspace. However, the transformation induces a direct coupling between the molecular electronic level and the bosonic environment in the last term in Eq. (12). An additional transformation is applied to remove this coupling, which is given by

$$S_2 = 2 d^\dagger d \sum_\beta \frac{\lambda\Lambda_\beta}{\hbar^2 \Omega\omega_\beta} \left(b_\beta - b_\beta^\dagger\right). \tag{16}$$

As a result, the double-transformed Hamiltonian reads

$$\begin{aligned}\overline{\overline{H}} = e^{S_2}\overline{H}e^{S_2^\dagger} = \bar{\bar{\epsilon}}d^\dagger d + \hbar\mathit{\Omega} a^\dagger a + 4d^\dagger d\sum_\beta \frac{\lambda \mathit{\Lambda}_\beta^2}{\hbar^2\mathit{\Omega}\omega_\beta}\left(a+a^\dagger\right) \\ + \sum_{k\in \mathrm{S/T}} \epsilon_k c_k^\dagger c_k + \sum_\beta \hbar\omega_\beta b_\beta^\dagger b_\beta + \sum_\beta \mathit{\Lambda}_\beta \left(a+a^\dagger\right)\left(b_\beta + b_\beta^\dagger\right) \\ + \sum_{k\in \mathrm{S/T}} \left(V_k XY c_k^\dagger d + V_k^* Y^\dagger X^\dagger d^\dagger c_k\right). \end{aligned} \tag{17}$$

In the above expression, we have introduced

$$\bar{\bar{\epsilon}} = \bar{\epsilon} - 4\sum_\beta \frac{\lambda^2\mathit{\Lambda}_\beta^2}{\hbar^3\mathit{\Omega}^2\omega_\beta}, \tag{18}$$

$$Y = \prod_\beta \exp\left(\frac{2\lambda\mathit{\Lambda}_\beta}{\hbar^2\mathit{\Omega}\omega_\beta}\left(b_\beta^\dagger - b_\beta\right)\right). \tag{19}$$

The Hamiltonian in Eq. (17) again contains a direct coupling between electronic and vibrational degrees of freedom. For a weak coupling between the vibration and bosonic bath, however, the interaction strength of the direct coupling term in the Hamiltonian in Eq. (17) is weaker than in the original Hamiltonian in Eq. (9). By repeatedly applying similar transformations, we can successively remove this term. Eventually, we obtain the Hamiltonian given by

$$\overline{H} = \overline{H}_\mathrm{S} + \overline{H}_\mathrm{E} + \overline{H}_\mathrm{SE}, \tag{20}$$

$$\overline{H}_\mathrm{S} = \bar{\epsilon}d^\dagger d + \hbar\mathit{\Omega} a^\dagger a, \tag{21}$$

$$\overline{H}_\mathrm{E} = H_\mathrm{F} + H_\mathrm{B} = \sum_{k\in \mathrm{S/T}} \epsilon_k c_k^\dagger c_k + \sum_\beta \hbar\omega_\beta b_\beta^\dagger b_\beta, \tag{22}$$

$$\overline{H}_\mathrm{SE} = \sum_{k\in \mathrm{S/T}} \left(V_k X c_k^\dagger d + V_k^* X^\dagger d^\dagger c_k\right) + \sum_\beta \mathit{\Lambda}_\beta \left(a^\dagger + a\right)\left(b_\beta^\dagger + b_\beta\right). \tag{23}$$

Based on the model system described by the Hamiltonian in Eq. (20), we employ a Born-Markov master equation approach [5, 10, 8, 6] in combination with the secular approximation

[1] to calculate the conductance-voltage characteristics. This method treats the molecular dynamics within second-order in the molecule-environment coupling. Within this formalism, the reduced density matrix is determined by the following equation of motion:

$$\frac{\partial \rho(t)}{\partial t} = -\, i \left[\overline{H}_{\mathrm{S}}, \rho(t) \right] - \int_0^\infty \mathrm{Tr}_{\mathrm{B+F}} \left\{ \left[\overline{H}_{\mathrm{SE}}, \left[e^{-i\tau(\overline{H}_{\mathrm{S}}+H_{\mathrm{E}})} \overline{H}_{\mathrm{SE}} e^{i\tau(\overline{H}_{\mathrm{S}}+H_{\mathrm{E}})}, \rho(t)\rho_{\mathrm{E}} \right] \right] \right\} \mathrm{d}\tau. \tag{24}$$

Here, $\mathrm{Tr}_{\mathrm{B+F}}$ denotes the trace over environmental bosonic and fermionic degrees of freedom and ρ_{E} the equilibrium density matrix of the environments given by

$$\rho_{\mathrm{E}} = \rho_{\mathrm{F}} \rho_{\mathrm{B}} = Z^{-1} e^{-(H_{\mathrm{F}} - \mu_{\mathrm{T}} N_{\mathrm{T}} - \mu_{\mathrm{S}} N_{\mathrm{S}})/(k_{\mathrm{B}} T_{\mathrm{F}})} e^{-H_{\mathrm{B}}/(k_{\mathrm{B}} T_{\mathrm{B}})}, \tag{25a}$$

$$Z = \mathrm{Tr}_{\mathrm{F}} \left\{ e^{-(H_{\mathrm{F}} - \mu_{\mathrm{T}} N_{\mathrm{T}} - \mu_{\mathrm{S}} N_{\mathrm{S}})/(k_{\mathrm{B}} T_{\mathrm{F}})} \right\} \mathrm{Tr}_{\mathrm{B}} \left\{ e^{-H_{\mathrm{B}}/(k_{\mathrm{B}} T_{\mathrm{B}})} \right\}. \tag{25b}$$

Here, $N_{\mathrm{S}} = \sum_{k \in \mathrm{S}} c_k^\dagger c_k$ ($N_{\mathrm{T}} = \sum_{k \in \mathrm{T}} c_k^\dagger c_k$) is the occupation number operator of the substrate (tip).

As an important observable to describe charge transport, we introduce the electrical current

$$I_K(t) = 2ie \int_0^\infty \mathrm{Tr}_{\mathrm{B+F+S}} \left\{ \left[e^{i\tau(\overline{H}_{\mathrm{S}}+H_{\mathrm{E}})} \overline{H}_{\mathrm{SE}} e^{-i\tau(\overline{H}_{\mathrm{S}}+H_{\mathrm{E}})}, \rho(\tau)\rho_{\mathrm{E}} \right] N_K \right\} \mathrm{d}\tau, \tag{26}$$

where Tr_{S} denotes the trace over system degrees of freedom. For the derivation of Eq. (26), the same approximations were used as for Eq. (24).

The parameters determined by the fitting procedure are summarized in Tab. 2.

Supplementary Table 2: Overview of the parameters obtained from the fitting scheme for the conductance-voltage characteristics in Figure 3a for $Z = 0$. The corresponding conductance-voltage characteristics is depicted in Figure 4b.

Γ_{T} (neV)	Γ_{S} (neV)	λ/Ω	Ω (meV)	γ
100	580	2.4	20	0.1

Supplementary references

1. Blum, K. *Density matrix theory and applications* (Springer Science & Business Media, 2012).

2. Braig, S. & Flensberg, K. Vibrational sidebands and dissipative tunneling in molecular transistors. *Phys. Rev. B* **68,** 205324 (2003).

3. Čížek, M., Thoss, M. & Domcke, W. Theory of vibrationally inelastic electron transport through molecular bridges. *Phys. Rev. B* **70,** 125406 (2004).

4. Galperin, M., Nitzan, A. & Ratner, M. A. Resonant inelastic tunneling in molecular junctions. *Phys. Rev. B* **73,** 045314 (2006).

5. Harbola, U., Esposito, M. & Mukamel, S. Quantum master equation for electron transport through quantum dots and single molecules. *Phys. Rev. B* **74,** 235309 (2006).

6. Härtle, R. & Thoss, M. Resonant electron transport in single-molecule junctions: Vibrational excitation, rectification, negative differential resistance, and local cooling. *Phys. Rev. B* **83,** 115414 (2011).

7. Haupt, F., Cavaliere, F., Fazio, R. & Sassetti, M. Anomalous suppression of the shot noise in a nanoelectromechanical system. *Phys. Rev. B* **74,** 205328 (2006).

8. Lehmann, J., Kohler, S., May, V. & Hänggi, P. Vibrational effects in laser-driven molecular wires. *J. Chem. Phys.* **121,** 2278–2288 (2004).

9. Mahan, G. D. *Many-particle Physics* (Springer Science & Business Media, Berlin, 2013).

10. Mitra, A., Aleiner, I. & Millis, A. J. Phonon effects in molecular transistors: Quantal and classical treatment. *Phys. Rev. B* **69,** 245302 (2004).

11. Nazin, G. V., Wu, S. W. & Ho, W. Tunneling rates in electron transport through double-barrier molecular junctions in a scanning tunneling microscope. *Proc. Natl. Acad. Sci. U.S.A.* **102,** 8832–8837 (2005).

12. Wilkins, R., Ben-Jacob, E. & Jaklevic, R. C. Scanning-tunneling-microscope observations of Coulomb blockade and oxide polarization in small metal droplets. *Phys. Rev. Lett.* **63,** 801–804 (1989).
